# Benchmarking genomic foundation models for binary classification of gene fusion breakpoints from DNA sequences

**DOI:** 10.1186/s13040-026-00553-1

**Published:** 2026-04-06

**Authors:** Radim Krupička, Mariana Komárková, Bohuslav Dvorský, Kateřina Kollinová, Ondřej Klempíř

**Affiliations:** 1 Department of Biomedical Informatics, Faculty of Biomedical Engineering, Czech Technical University in Prague, nám. Sítná 3105, Kladno, Czech Republic; 2 Institute of Hematology and Blood Transfusion, Prague, Czech Republic

**Keywords:** Genomic foundation models, Gene fusion classification, DNA sequence analysis, Transformer models, Nucleotide Transformer, Evo2, Bioinformatics benchmarking

## Abstract

**Background:**

Gene fusions are critical drivers of oncogenesis and diagnostic biomarkers in various cancers. However, their detection from RNA or DNA sequencing, when performed using traditional analytical methods, encounters challenges related to sample quality, computational complexity, and noise. Although deep learning is more robust, it usually requires large labeled datasets and substantial training resources. Genomic foundation models (GFMs), which are pre-trained on pangenome-scale data, offer a promising solution to these issues.

**Methods:**

This study presents the first comprehensive benchmark of four transformer-based GFMs, Nucleotide Transformer (NT), Evo2, HyenaDNA, and DNABERT2, for the classification of gene fusion breakpoints. Using the curated FusionAI dataset of ~ 52,000 sequences, we extracted embeddings from 10-kilobase-pair (kbp) DNA sequences surrounding fusion breakpoints. We evaluated the quality of these representations qualitatively using t-SNE visualization and quantitatively by training lightweight classifiers (Support Vector Machines and simple Neural Networks) on the fixed embeddings.

**Results:**

NT achieved the best performance with an accuracy of 0.967 and an F1 score of 0.967. This result outperformed the dedicated deep learning baseline (FusionAI, with an accuracy of 0.894). Evo2 was the second-best performer (accuracy: 0.920), demonstrating robustness derived from evolutionary pretraining. Conversely, DNABERT2 failed to compete (accuracy 0.677–0.723). Furthermore, sample efficiency analysis revealed that NT required only ~ 2,600 samples to reach 95% of its peak performance, whereas the baseline required over 14,000 samples.

**Conclusions:**

These findings demonstrate that advanced GFMs, particularly the NT and Evo2 models, generate highly discriminative ‘out-of-the-box’ embeddings. These embeddings significantly outperform dedicated deep learning baselines while requiring a fraction of the training data and computational time. This suggests that GFMs could be a scalable, data-efficient way of developing precise genomic diagnostic tools, particularly for rare diseases.

## Introduction

Gene fusions are genetic alterations that typically arise from large-scale structural changes in the DNA, leading to the joining of two previously non-adjacent genes [1, 2]. These fusions can result in the production of abnormal proteins or cause dysregulation of gene expression. Aberrant gene fusions are implicated in the pathogenesis of various cancers, including hematologic malignancies such as leukemia, as well as numerous solid tumors. They serve as important diagnostic, prognostic, and therapeutic biomarkers. Fusion events are most commonly detected through RNA sequencing due to its ability to capture expressed fusion transcripts. However, DNA sequencing can also be used, albeit less frequently, due to its higher cost and lower sensitivity to expressed fusions [3]. Nevertheless, both approaches face significant technical challenges, including degraded RNA samples, variability in library preparation, high computational demands, and data noise — all of which can contribute to both false positives and false negatives [4, 5].

Standard bioinformatic tools, such as Arriba [6] and STAR-Fusion [7], have been widely adopted to address these analytical challenges. While these tools offer high specificity, they often lack robustness and generalizability when dealing with variable or low-quality data [7, 8]. Additionally, these tools require expert-driven parameter tuning to perform optimally [9], which hinders their scalability for large research cohorts and clinical applications [10]. Simultaneously, while emerging technologies like long-read RNA sequencing offer new opportunities to resolve full-length fusion structures, they introduce their own challenges, such as high intrinsic error rates, which require specialized structural algorithms like the splicing-graph-based framework GFSeeker [11].

The overarching limitations of both traditional and specialized algorithmic approaches have driven the current trend toward machine learning (ML) and deep learning (DL) models, which aim to reliably extract fusion signals from complex, noisy data with minimal manual intervention [12–14]. However, developing DL models traditionally presents significant challenges, including the need for substantial labeled training data and substantial domain expertise. These are common bottlenecks in genomics [15, 16].

The recent emergence of genomic foundation models (GFMs), which are based on large language model architectures, offers a potential solution [17]. GFMs are pre-trained on vast, pangenome-scale datasets and can be fine-tuned for specific downstream tasks. They reportedly achieve high accuracy even with limited data.

A pioneering example is DNABERT [18], which adapts the BERT (Bidirectional Encoder Representations from Transformers) architecture to genomic sequences. DNABERT has demonstrated strong performance in predicting promoter regions, splice sites, and transcription factor binding sites. Building on this foundation, subsequent studies have fine-tuned or extended the model for various tasks. These include msBERT-Promoter [19], a model for DNA promoter identification and strength estimation; PLANNER [20], a model for predicting origins of replication sites; BERT-TFBD [21], and MutBERT [22].

A more recent and versatile model family is the Nucleotide Transformer [23], designed to predict molecular phenotypes directly from DNA sequences. Trained on 3,202 diverse human genomes and 850 non-human genomes, it leverages multi-task learning and transfer learning to overcome limitations posed by scarce annotated data. Its applications include functional element detection, chromatin accessibility analysis, and variant prioritization. Emerging models also target specific biological domains. For example, the Genetic Transformer [24] identifies causal variants in rare diseases, while HyenaDNA [25] captures long-range dependencies and processes DNA sequences up to one million nucleotides long. The Evo2 model [26] extends HyenaDNA’s capabilities by incorporating evolutionary conservation data into its architecture. This enables better prediction of the functional impact of genetic variants by improving the modeling of sequence conservation and long-range dependencies. While these models show great promise across various genomic tasks, their application to gene fusion detection remains unexplored.

Because pre-trained GFMs encode rich biological features into vector representations, we hypothesize that these embeddings can be used as inputs for a simple classifier that can distinguish between fusion-positive and fusion-negative sequences. This approach may offer improved accuracy and training efficiency while requiring significantly less labeled data. To test this hypothesis, this article compares the performance of current foundation models. We used the dataset generated from the FusionAI study [12] (previously used for deep learning) to explore whether GFMs could improve performance on this existing benchmark.

## Methods

In this study, the GFMs were used solely as pre-trained feature extractors with frozen weights; no parameters within the foundation models were updated during the experiments. Training was restricted exclusively to the lightweight downstream classification heads (SVM and NN) to map these fixed embeddings to the target classes.

For model evaluation, we used the dataset introduced and described in FusionAI study [12], comprising approximately 26,000 fusion-positive and 26,000 fusion-negative sequences. From the total of ~ 52,000 sequences, we used ~ 36,000 (~ 18,000 positive and ~ 18,000 negative) for training, identical to the training set in the original study. The remaining ~ 16,000 sequences were evenly divided into validation (~ 8,000) and test sets (~ 8,000), while preserving the same ratio of positive and negative samples. We kept all data partitions consistent across experiments to ensure a fair comparison between models.

Each data sample consisted of a 10 kbp sequence surrounding the fusion breakpoint in gene 1 (sequence 1) and gene 2 (sequence 2). Each sequence was encoded using four widely used genomics foundation models based on the transformer architecture: Nucleotide Transformer [23], HyenaDNA [25], Evo2 [26], and DNABERT2 [18]. The selection of these four GFMs was designed to provide a representative cross-section of current genomic modeling architectures. The Nucleotide Transformer represents high-capacity transformer encoders trained on multi-species pangenomes. Evo2 (7B) serves as a benchmark for large-scale hybrid architectures incorporating evolutionary conservation. HyenaDNA represents sub-quadratic models optimized for ultra-long contexts, while DNABERT2 represents efficient BPE-based models widely used for local motif analysis. Embeddings were extracted from hidden states using author-recommended layer configurations to ensure optimal performance. Table 1 provides a systematic comparison of model capacities, tokenization strategies, and specific layers. Implementation details for each GFM follow.

### Nucleotide Transformer (NT)

This model is specifically optimized for capturing high-level sequence patterns and predicting molecular phenotypes, such as splicing and regulatory elements. For our benchmark, we used the 500M_multi_species_v2 model. The NT was configured with a maximum sequence length of 1,671 tokens, and 1024-dimensional embeddings were extracted from the 20th transformer layer (out of 24 total layers). Given the model’s 6-mer tokenization scheme, this creates a receptive field of 10,026 bp, which fully encompasses the 10 kbp input sequences centered on the gene fusion breakpoints. This configuration allowed the model to process the entire region of interest in a single pass without the need for truncation or sliding window strategies.

### Evo2 (Evo)

Designed for large-scale genome modeling and clinical variant interpretation, Evo2 incorporates evolutionary conservation data across all domains of life. We utilized the evo2_7b checkpoint for sequence encoding. Through byte-level tokenization, each character in the DNA string was converted to its corresponding UTF-8 integer value. Embeddings were subsequently extracted from the blocks.28.mlp.l3 internal layer, where the embedding dimension was 4096. Unlike k-mer-based approaches, Evo2 utilizes a byte-level tokenizer where each nucleotide maps directly to a single token, preserving single-base resolution. Leveraging the model’s StripedHyena architecture designed for long-range genomic modeling, we processed the input sequences at their full 10 kbp length (10,000 tokens) without truncation or downsampling.

### HyenaDNA (Hyena)

This architecture is designed to handle long-range dependencies and structural genomic changes, such as the formation of extrachromosomal circular DNA (eccDNA). The “hyenadna-large-1m-seqlen-hf” model was used with 1 million token indices, which employs a character-level tokenizer where each nucleotide corresponds to a single token. HyenaDNA’s sub-quadratic operator allowed for efficient processing of the full input at single-nucleotide resolution without the need for downsampling or token aggregation. Embeddings were extracted from the model’s final hidden layer, where the embedding dimension was 256.

### DNABERT2 (BERT)

Widely utilized for local motif analysis, including the prediction of promoters and transcription factor binding sites, DNABERT2 adapts the BERT architecture to genomic sequences. The zhihan1996/DNABERT-2-117 M model was utilized. DNABERT-2 employs Byte Pair Encoding (BPE), which results in variable token counts for fixed-length DNA sequences. To enable consistent batch processing and preserve spatial alignment, we standardized all input tensors to a fixed length of 2,143 tokens. This threshold was empirically determined to accommodate the maximum tokenized length of any 10 kbp sequence in our dataset. We implemented a symmetric padding strategy, adding special tokens to both ends of shorter sequences. DNA sequences were tokenized into overlapping k-mers using the model’s specific tokenizer. The final embeddings were then extracted from the hidden states of the last transformer layer, where the embedding dimension was 768.

**Table 1:** Systematic comparison of the architectural and computational characteristics of the four evaluated Genomic Foundation Models (GFMs)

	NT	Evo2	HyenaDNA	DNABERT2
Architecture	Transformer Encoder	StripedHyena 2	Hyena operator	Transformer Encoder
Focus / Strengths	Regulatory patterns & splicing	Evolutionary conservation	Long-range dependencies	Local motifs & TF binding
Pre-training Data	3,202 human + 850 non-human genomes	OpenGenome2 (all domains of life)	Human reference genome (Hg38)	Genomes from 135 species
Parameters	500 M	7B	54.6 M	117 M
Max Context Length (Approximate)	10k bp	1 M bp	1 M bp	10k bp
Tokens	Fixed k-mers (6 bp)	Single Nucleotide	Single Nucleotide	Variable-length subword tokens (1–6 bp)
Default (used) embedding layer	Layer 20	blocks.28.mlp.l3	Final hidden layer	Last transformer layer
Embedding dimension	1024	4096	256	768
Scaling Behavior	Quadratic	Sub-quadratic	Near-linear	Quadratic
VRAM (batch size = 1)*	~ 4 GB	~ 19 GB	~ 2 GB	~ 3 GB
Latency (ms/sample)*	~ 45 ms	~ 280 ms	~ 10 ms	~ 25 ms

* Inference latency (ms/sample) and peak VRAM usage (GB) were tested on a computing cluster node equipped with 1x NVIDIA H100 (96GB), dual AMD EPYC 9454 48-Core Processors, and 128 GB of RAM

From each sequence, we primarily extracted the middle embedding, which corresponds to the fusion breakpoint. Due to the contextual nature of the embeddings, this central embedding also encodes information from the surrounding sequence. We concatenated the middle embeddings from both sequences and used the resulting vector for classification.

To ensure a comprehensive benchmark and address the limitations of localized extraction, we implemented a mean pooling strategy for comparison. This approach calculates the average embedding across all base pairs in the 10 kbp input sequence. We introduced this strategy to evaluate the models’ ability to capture the global genomic context and mitigate positional noise, especially for models using Byte Pair Encoding (BPE), such as DNABERT2. In these models, variable token lengths can cause the mathematical “middle” token to shift away from the biological breakpoint. Evaluating both the middle and mean embeddings provides a more rigorous assessment of how different GFM architectures handle structural variations and positional uncertainty.

We visualized embedding quality using t-distributed Stochastic Neighbor Embedding [27] (t-SNE) with perplexity = 30 and 1,000 iterations on a random subset of 1,000 samples. Class separability was qualitatively assessed by visual inspection of the 2D projections.

### Classification

**Fig. 1:**
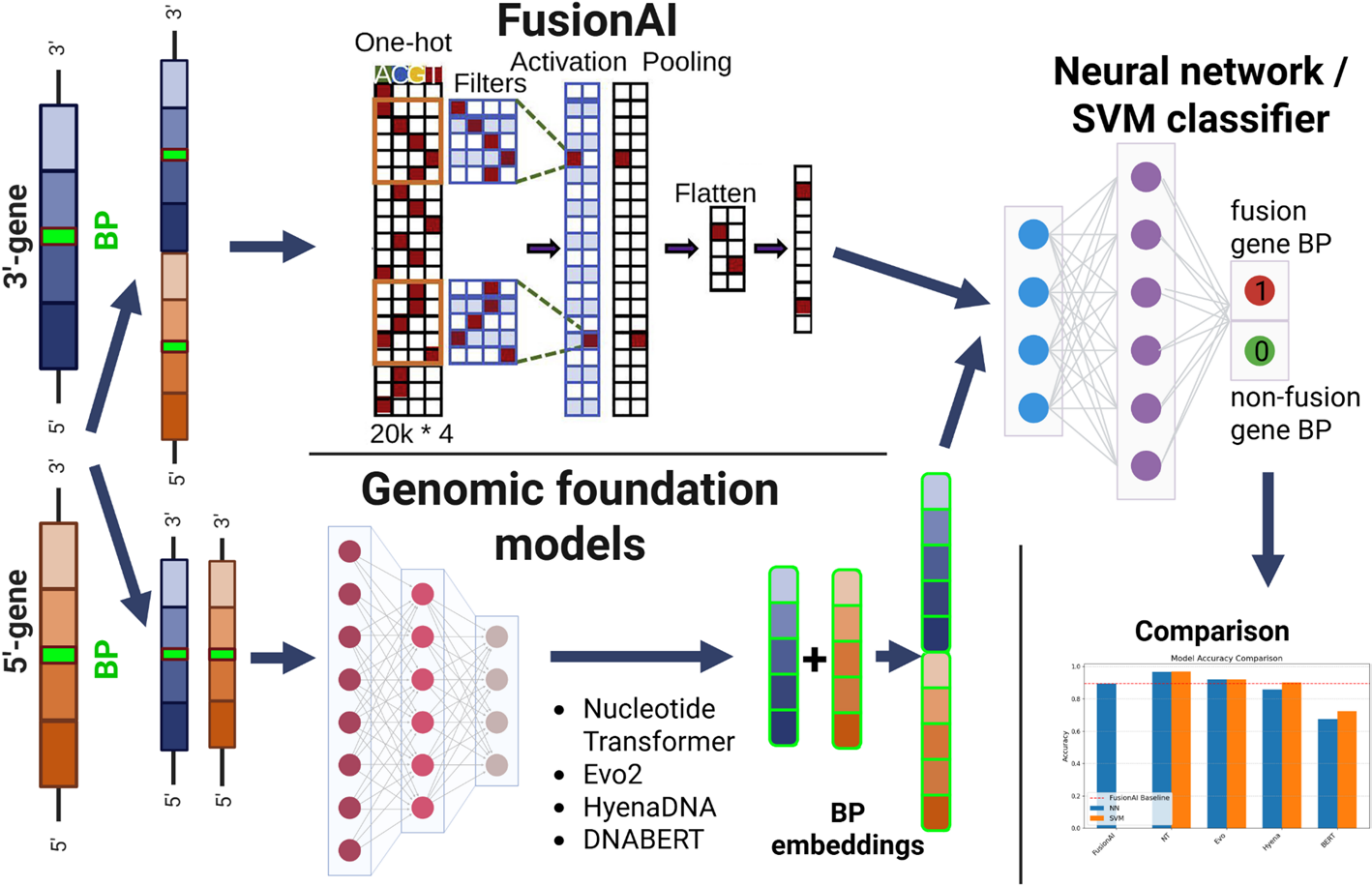
Diagram comparing FusionAI and Genomic Foundation Models for gene fusion breakpoint (BP) classification

For classification, we used two classifiers following the FusionAI article [12] : (i) a support vector machine (SVM) with RBF kernel with C = 1.0 and γ automatically computed as 1/(n_features × variance). Hyperparameters were not tuned via cross-validation; fixed values were used across all experiments. (ii) a fully connected neural network (NN) with an architecture adopted from FusionAI article [12]. We reimplemented the entire CNN architecture as described in FusionAI [12] to allow for a direct comparison (see Fig. 1). The classifier architecture consists of a single-layer feedforward neural network with one hidden layer containing 32 neurons with ReLU activation, followed by dropout (*p* = 0.4) and a softmax output layer for binary classification. The model was compiled using the Adadelta optimizer with categorical cross-entropy loss. Training was performed with a batch size of 256. The neural networks were trained for up to 1000 epochs to ensure training stability and convergence.

### Evaluation metrics

We evaluated model performance on the test set using accuracy (percentage of correct predictions), precision (weighted average across classes), recall (weighted average across classes), F1 score (weighted average), and area under the ROC curve (AUC-ROC, macro-averaged for multi-class). For neural networks, we report the final epoch performance; for SVMs, we report the single training run results.

### Sample efficiency

To assess sample efficiency, we trained models on 19 stratified training subsets ranging from 200 to 36,302 samples. We fit logarithmic functions (y = a·log(x) + b) to the resulting learning curves and computed: (1) the sample size required to reach 95% of final accuracy (Samples@95%) by inverting the fitted curve: x₉₅ = exp((0.95·y_final - b) / a), and (2) a logarithmic efficiency score defined as (Efficiency@95%) y_final / ln(x₉₅), which quantifies accuracy achieved per natural logarithm unit of training data. Higher efficiency scores indicate models that reach high performance with logarithmically fewer samples. Accuracy values were normalized to 0-100% of each model’s maximum performance for cross-model visualization.

### Robustness testing

To evaluate the resilience of the models to noise and artifacts typical of real-world sequencing (e.g. variable coverage or alignment errors), we performed a sensitivity analysis on a subset of 800 samples from the test set. We introduced two types of perturbation: Random point mutations: Simulating sequencing noise by randomly mutating 1–32% of nucleotides; 2. Positional shifts: Simulating uncertainty in breakpoint localization by shifting sequences by 1 to 64 bp. These tests were conducted for both the “middle embedding” (representing the pre-localized breakpoint) and the “mean embedding” (averaging across all sequence tokens) strategies.

### Implementation details

All models were implemented in Python 3.11 using Keras 3.0 with PyTorch backend. Neural network training used the Adadelta optimizer with default Keras parameters (learning_rate = 1.0, rho = 0.95, epsilon=1e-07). SVM models were trained using scikit-learn 1.4 with default parameters unless otherwise specified. Random sampling and data splitting used a fixed seed (42) to ensure reproducibility across different training set sizes. All model training and inference benchmarking were conducted on a high-performance computing cluster node equipped with an NVIDIA H100 GPU (96 GB VRAM), dual AMD EPYC 9454 48-Core Processors, and 128 GB of system RAM. All source code and notebooks with results used for comparison are available at https://github.com/kbi-fbmi/articles--2026fusionEmbBenchmark/. The data and results files are available on Zenodo [28].

## Results

### Visual assessment of embedding quality

**Fig. 2:**
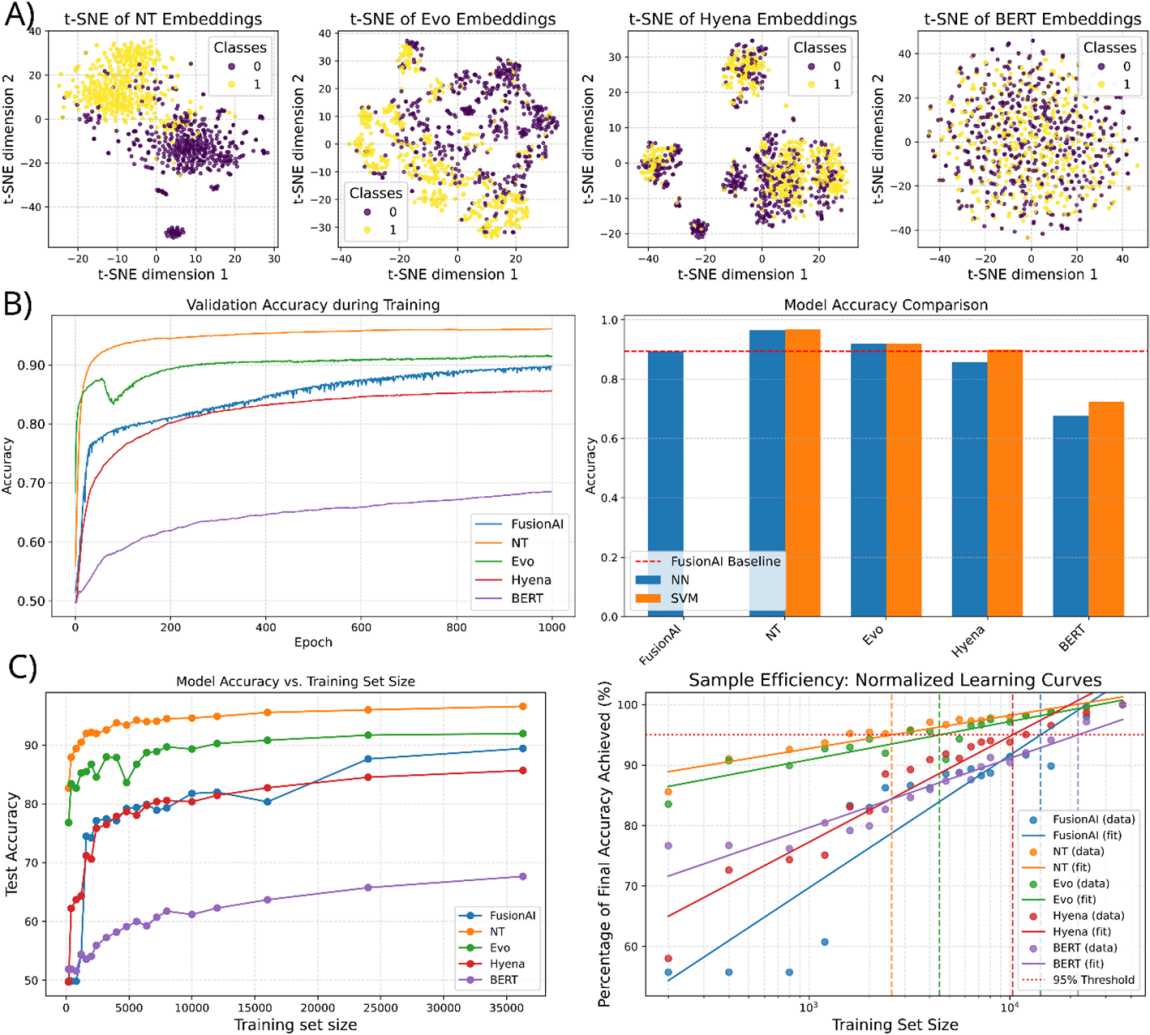
Comparative analysis of middle embedding models versus a reimplemented FusionAI classifier for gene fusion classification (**A**) t-SNE visualization of embedding spaces: class separability of fusion-positive and fusion-negative sequences across genomic foundation models. (**B**) Training dynamics (left) and comparative accuracy (right). (**C**) Sample efficiency analysis: Scaling of model accuracy with training data size (left) and log-transformed normalized learning curves with fitted regression lines indicating the 95% convergence threshold (right)

We visualized the embeddings of fusion-positive and fusion-negative sequences using t-SNE to qualitatively assess whether genomic foundation models (GFMs) capture features relevant to gene fusion binary classification. The resulting projections revealed differences in class separability across the evaluated models (see Fig. 2A).

### Classification performance

We evaluated the efficacy of these embeddings using two classifiers, an SVM and a neural network, and compared them against the FusionAI baseline model, which is a dedicated deep learning protocol. Table 2 summarizes the results on the full test set (~ 8 K samples), and Fig. 2B shows training convergence.

- **Nucleotide Transformer** achieved the highest overall performance, significantly outperforming the baseline. Using the SVM classifier, NT achieved an accuracy of 0.967 and an F1 score of 0.967, whereas **FusionAI** achieved an accuracy of 0.894. The neural network classifier yielded nearly identical results for NT (accuracy: 0.966; ROC AUC: 0.994), demonstrating the robustness of these embeddings regardless of the classification method.
- **Evo2** was the second-best performer, consistently surpassing the FusionAI baseline. Both the support vector machine (SVM) and neural network (NN) classifiers achieved an accuracy and F1 score of 0.920 and a ROC AUC of 0.970–0.975.
- **HyenaDNA** produced mixed results. When paired with a simple neural network, its performance was lower than the baseline (accuracy: 0.857 vs. 0.894). However, using an SVM improved HyenaDNA’s performance, achieving an accuracy of 0.900 and slightly surpassing the FusionAI baseline.
- **DNABERT2** failed to compete with the other foundation models or the baseline. Its accuracy ranged from 0.677 (NN) to 0.723 (SVM), and its ROC AUC was significantly lower at 0.745–0.799.

**Table 2:** Comparative performance of embedding models versus the reimplemented FusionAI baseline on the full test set

Model	Classifier	Accuracy	Precision	Recall	F1 Score	ROC AUC
FusionAI	NN	0.894	0.894	0.894	0.894	0.960
NT	**NN (middle)**	**0.966**	**0.966**	**0.966**	**0.966**	**0.994**
	NN (mean)	0.893	0.893	0.893	0.893	0.958
	**SVM**	**0.967**	**0.972**	**0.962**	**0.967**	**0.995**
Evo2	NN (middle)	0.920	0.920	0.920	0.920	0.970
	NN (mean)	0.879	0.879	0.879	0.879	0.948
	SVM	0.920	0.920	0.920	0.920	0.975
HyenaDNA	NN (middle)	0.857	0.858	0.857	0.857	0.936
	NN (mean)	0.777	0.777	0.777	0.777	0.852
	SVM	0.900	0.880	0.925	0.902	0.962
DNABERT2	NN (middle)	0.677	0.678	0.677	0.676	0.745
	NN (mean)	0.726	0.748	0.726	0.720	0.819
	SVM	0.723	0.737	0.690	0.713	0.799

### Robustness testing

**Fig. 3:**
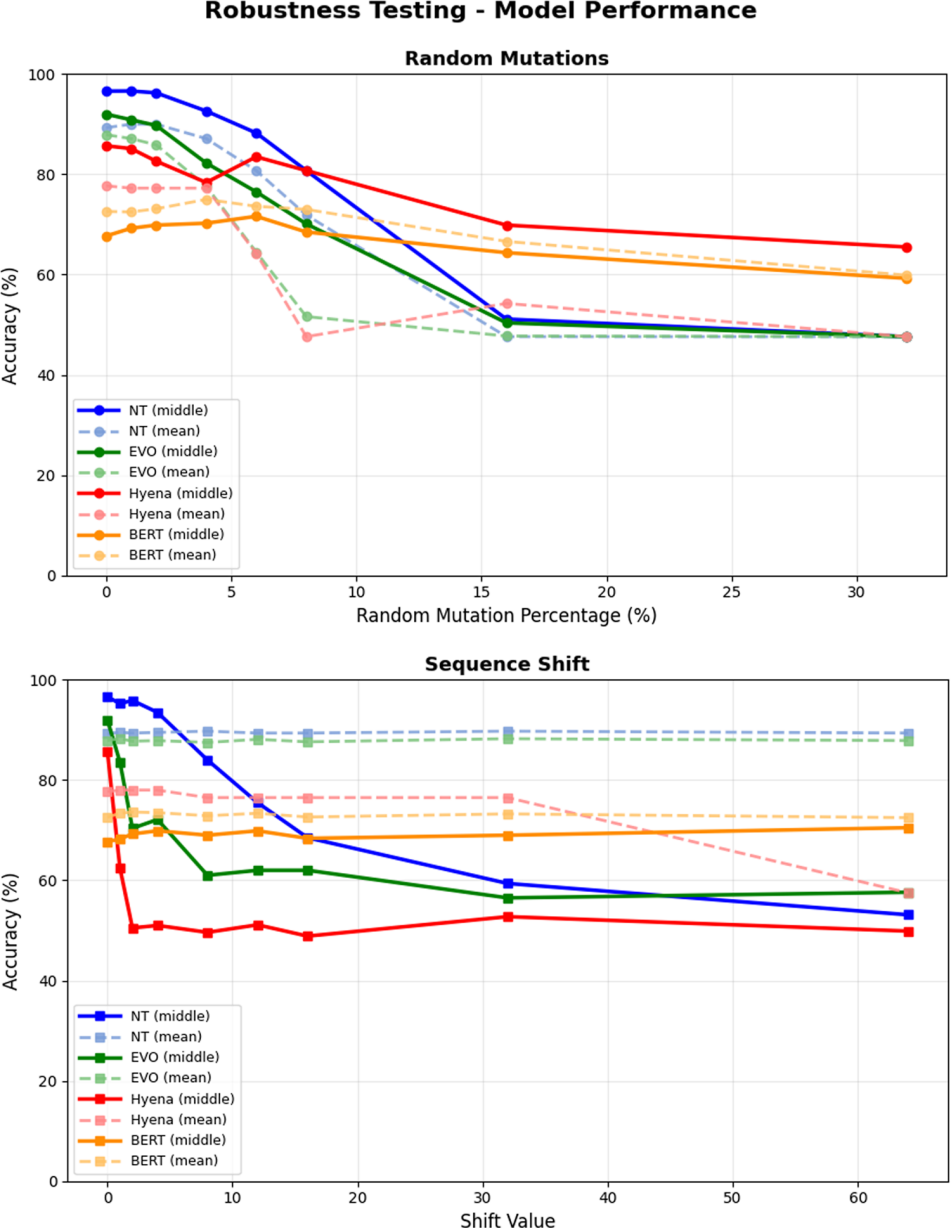
Robustness analysis of GFM embeddings. Evaluation of model resilience against synthetic sequence perturbations. (Left) Accuracy under random point mutations (1–32%). Solid lines denote “middle embedding”; dashed lines denote “mean embedding” (global). (Right) Accuracy under positional sequence shifts (1–64 bp). Tests conducted on a random subset of 800 test samples

The sensitivity analysis is plotted in Fig. 3 and shows that the NT with middle embedding maintains the highest accuracy at low mutation rates but experiences a sharp decline when random mutations exceed 8%. In contrast, the mean embedding strategies for all models, represented by dashed lines, exhibit a flatter response to both random mutations and sequence shifts. Specifically, while the middle embedding performance for NT and Evo2 drops significantly when the sequence is shifted by more than 8 bp, the mean embedding accuracy remains near-constant across all tested shift values up to 64 bp. For DNABERT2, the mean embedding consistently outperformed the middle embedding across the entire range of perturbations.

### Computational efficiency

A major advantage of the GFM-based approach observed in this study is the dramatic reduction in training time. The original FusionAI protocol required approximately 40 h to train for 1,000 epochs to ensure stability. In contrast, the foundation model workflow, comprising embedding extraction and training of the lightweight classifiers, was completed in under 10 min.

#### Sample efficiency

To evaluate how well the models perform in data-scarce regimes, we analyzed learning curves across training subsets ranging from 200 to ~ 36,000 samples. We calculated the number of samples required to reach 95% of the model’s final accuracy (Samples@95%) and a logarithmic efficiency score (see Table 3; Fig. 2C).

- **Nucleotide Transformer** demonstrated superior sample efficiency, requiring only 2,581 samples to reach 95% of its peak performance (Efficiency Score: 12.29).
- **Evo2** followed, requiring 4,461 samples (Efficiency Score: 10.94).
- **HyenaDNA** required 10,303 samples, approaching the data requirements of the baseline.
- **FusionAI** (Baseline) required 14,200 samples to reach its convergence threshold.
- **DNABERT2** was the least efficient, requiring 21,768 samples to stabilize its (comparatively lower) performance.

**Table 3:** Data efficiency benchmark comparing embedding models to the reimplemented FusionAI baseline (trained for 1,000 epochs)

Model	Samples (at 95%)	Accuracy (at 95%)	Efficiency Score (at 95%)
FusionAI	14,200	0.849	9.35
**NT**	**2**,**581**	**0.918**	**12.29**
**Evo2**	**4**,**461**	**0.874**	**10.94**
HyenaDNA	10,303	0.814	9.27
DNABERT2	21,768	0.643	6.78

## Discussion

To the best of our knowledge, this study presents the first comprehensive benchmark of genomic foundation models for gene fusion classification in DNA sequences. Our results show that using pre-trained embeddings from advanced GFMs, such as NT and Evo2, significantly improves accuracy compared to dedicated deep learning protocols like FusionAI, while requiring a fraction of the computational resources and training data.

The Nucleotide Transformer produced the most distinct class separation, forming dense, non-overlapping clusters of fusion-positive and fusion-negative sequences. This high-quality representation resulted in superior classification performance (accuracy: 0.967), which remained consistent regardless of whether a simple support vector machine (SVM) or neural network was used as the classification head. Implementing the mean pooling strategy confirmed that, for the highest-performing models (particularly NT), localized features at the breakpoint are paramount. The observed decline in accuracy (up to 10% for NT) indicates that averaging across the entire 10 kbp sequence dilutes the specific signal of the fusion event by including irrelevant genomic context.

Evo2 followed closely behind NT, consistently surpassing the baseline and demonstrating that models incorporating evolutionary information are highly effective at characterizing structural genomic events. In terms of sequencing noise, the robustness of EVO2 and NT is noteworthy; both models maintained high accuracy up to a random mutation rate of 8%, suggesting high reliability for clinical diagnostics where error rates are typically much lower [29, 30].

These results were already qualitatively suggested by the t-SNE visualizations (Fig. 2A), where NT and Evo2 exhibited the most pronounced class separation. However, such projections must be interpreted with caution. As t-SNE is a non-linear technique optimized for local neighborhood structures, the distances between clusters do not necessarily reflect true biological variance or the global relationship between samples. To ensure robustness, we verified these patterns across varying perplexity values (10–50), which yielded consistent clustering topologies.

Our analysis further revealed that models utilizing the middle embedding strategy exhibit high sensitivity to positional uncertainty. The classification stability for EVO2 and NT decreased when the sequence was shifted by 2 and 4 bp, respectively (see Fig. 3), suggesting that localized embeddings are highly dependent on precise breakpoint centering. Conversely, the mean embedding approach provided near-constant accuracy across the entire range of shifts (up to 64 bp), highlighting a clear trade-off between peak baseline precision and positional invariance.

A notable finding is that DNABERT2 was unable to compete in this specific benchmark. It showed significant class overlap and lower accuracy (accuracy: 0.677–0.723). However, mean pooling was the only strategy that produced competitive results for DNABERT2, with a score of 0.726. This indicates that its BPE-based tokenization is more sensitive to localized extraction. This contrasts with recent literature, in which DNABERT2 and its predecessor are successful backbones for various genomic tasks [19, 20, 22, 31]. For example, DNABERT2 has been adapted for viral lineage classification in ViralLM [32], bacterial genome decoding, and competing against protein language models in downstream protein tasks [33].

The performance gap between the evaluated models stems from key architectural differences. The superior precision of NT and Evo2 likely arises from their high parameter capacity and diverse pangenomic pre-training. Specifically, NT’s 6-mer tokenization captures localized regulatory patterns critical for breakpoint identification, while Evo2’s byte-level resolution allows for the detection of subtle structural motifs. In contrast, DNABERT2’s underperformance suggests that BPE may introduce positional noise; variable-length tokens can shift the mathematical center away from the biological breakpoint, diluting the signal. This discrepancy between DNABERT2’s success in literature and our findings highlights that its objectives, while excellent for semantic patterns in viral or bacterial genomes, may not produce sufficiently discriminative embeddings for human gene fusions without extensive fine-tuning. Our results thus indicate a clear trade-off: larger models (NT, Evo2) generate more linearly separable spaces for structural variations than models optimized for local motif analysis like DNABERT2.

HyenaDNA is designed to process context lengths up to one million tokens and showed mixed results. Although it outperformed the baseline when paired with an SVM, it did not achieve the same level of precision as NT or Evo2 in our “frozen embedding” setting. These results are similar to those from the NextVir study [34], which benchmarked GFMs for oncoviral classification. The NextVir authors also noted that base models provided decent results with simple adapters but that maximizing performance often required fine-tuning strategies, such as Low-Rank Adaptation (LoRA), particularly for Hyena-based models.

However, HyenaDNA’s potential for structural tasks remains strong. The HyenaCircle model [35] recently demonstrated that HyenaDNA-based architectures can predict eccDNA effectively from sequence data. Since eccDNA formation shares mechanistic similarities with gene fusions, which involve DNA breaks and re-ligation, it is likely that HyenaDNA captures the relevant signal. However, this signal is more complex for linear classifiers to disentangle than the representations produced by NT or Evo2. Furthermore, HyenaDNA’s single-nucleotide resolution has proven advantageous in viral taxonomy (e.g., ViTax) [36], as it can handle high mutation rates better than k-mer-based approaches can. Future work on gene fusions should explore fully tuning or using LoRA adapters to unlock HyenaDNA’s full potential for this task.

In comparison with RNA-based approaches, it is important to contextualize our work within the broader field of fusion classification. Recent machine learning efforts have primarily focused on identifying chimeric RNAs from RNA-seq data. For instance, transformer-based classifiers for chimeric reads have achieved success with DNABERT-based architectures [37]. However, our approach detects at the DNA level. This is crucial and distinct for clinical scenarios where RNA samples are degraded or unavailable. By replacing complex feature engineering with high-quality pretrained embeddings, we provide a more streamlined alternative that complements existing RNA-based methods.

### Computational and sample efficiency

A critical advantage of the GFM-based workflow is its dramatic increase in efficiency. Training time decreased from about 40 h for the FusionAI baseline to less than 10 min for our foundation model approach. Additionally, our analysis of sample efficiency underscores the “few-shot” capabilities of robust GFMs. NT required only ~ 2,500 samples to reach 95% of its peak performance, whereas the baseline required over 14,000 samples. This makes GFM-based approaches particularly promising for rare disease studies or clinical scenarios where large annotated datasets are scarce.

In conclusion, the landscape of genomic foundation models is rapidly expanding with the emergence of models like PathoLM [38] and Embed-Search-Align [39] for diverse tasks, and novel frameworks for unsupervised embedding evaluation [40]. However, our benchmark shows that NT and Evo2 provide the most robust “out-of-the-box” representations for human gene fusion classification. These models offer a superior balance of accuracy, speed, and data efficiency, paving the way for scalable, AI-driven genomic diagnostics.

Beyond training efficiency, the computational overhead of the embedding extraction phase is a critical factor for clinical scalability. As shown in our systematic comparison (Table 1), there is a significant trade-off between model capacity and resource requirements. While the 7B-parameter Evo2 model offers high resolution, it requires substantial hardware resources, with a peak VRAM usage of 19 GB and an inference latency of 280 ms per sample. In contrast, the 500 M-parameter NT achieves superior accuracy with significantly lower requirements (4 GB VRAM, 45 ms/sample). These metrics demonstrate that for high-throughput diagnostic workflows, smaller yet highly optimized foundation models like NT provide a more sustainable balance of performance and hardware accessibility.

### Limitations and future directions

To ensure a rigorous and reproducible comparison with established baselines, this study used the curated FusionAI benchmark dataset. This controlled environment enabled us to isolate the contribution of foundation model embeddings to classification performance rather than confounding the results with sequencing artifacts and variable coverage, which are often present in raw whole-genome sequencing (WGS) data. Although the current evaluation focused on binary classification to validate the discriminative power of these embeddings, this lays the groundwork for more granular structural analysis.

Another limitation of this study is its focus on “frozen” embeddings fed into lightweight classifiers. While this approach emphasizes computational efficiency and demonstrates GFMs’ robust “out-of-the-box” capabilities, it may not represent the models’ true performance ceiling. Recent research indicates that parameter-efficient fine-tuning (PEFT) techniques, such as low-rank adaptation (LoRA), can significantly improve model performance for specific genomic tasks by adapting the internal representations to the target domain. Future iterations of this work will explore the trade-off between the computational speed of frozen embeddings and the potentially higher accuracy achieved through LoRA fine-tuning, particularly for models like HyenaDNA, which may require task-specific adaptation to reach their full potential.

Building on these findings, our future work will extend this framework to precisely identify fusion breakpoints and type fusion partners. Breakpoint localization typically requires extensive annotated datasets to prevent overfitting, but our analysis of sample efficiency offers a promising approach. We demonstrated that models such as Nucleotide Transformer and Evo2 converge with significantly fewer samples than traditional baselines. This high learning efficiency suggests that training advanced heads for coordinate regression or token-level segmentation is computationally feasible, even with smaller, high-quality datasets available in clinical settings. For real-world deployment, we propose a localized pooling approach around the suspected breakpoint. Additionally, future research should focus on data augmentation with synthetic shifts to improve the models’ inherent resilience to alignment errors. Thus, we can bridge the gap between foundation models and precision diagnostics.

## Conclusion

This study presents the first comprehensive benchmark of genomic foundation models (GFMs) for the direct binary classification of gene fusions from DNA sequences. Our results show that using pre-trained embeddings from advanced models such as the Nucleotide Transformer and Evo2 significantly improves classification accuracy compared with specialized deep learning protocols such as FusionAI. Specifically, the Nucleotide Transformer achieved an accuracy of 0.967, followed by Evo2 with an accuracy of 0.920; both models outperformed the baseline accuracy of 0.894.

In addition to superior performance, the GFM-based approach offers dramatic computational efficiency. The training time was reduced from approximately 40 h required by the original protocol to under 10 min. Further analysis of sample efficiency highlighted the robustness of these models: the Nucleotide Transformer required only ~ 2,600 samples to reach 95% of its peak performance, whereas the baseline method needed over 14,000 samples. This characteristic is particularly valuable for clinical applications and rare disease research, where large annotated datasets are often unavailable.

Although certain models, such as DNABERT2, were unable to compete in this specific task, the overall findings confirm that GFMs offer a scalable and highly effective alternative to traditional methods. Future work should focus on extending these capabilities and integrating these efficient workflows into clinical genomic diagnostics.

## Data Availability

The datasets analyzed during the current study and learned models are available in the Zenodo repository: [https://zenodo.org/records/18713246]. The source code is available at [https://github.com/kbi-fbmi/articles--2026fusionEmbBenchmark].

## Abbreviations

ACC: Accuracy
AUC: Area Under the Curve
BERT: Bidirectional Encoder Representations from Transformers
BPE: Byte Pair Encoding
CNN: Convolutional Neural Network
DL: Deep Learning
DNA: Deoxyribonucleic acid
FN: False Negative
FP: False Positive
GFM: Genomic Foundation Model
MCC: Matthews Correlation Coefficient
ML: Machine Learning
NLP: Natural Language Processing
NN: Neural Network
NT: Nucleotide Transformer
SVM: Support Vector Machine
TN: True Negative
TP: True Positive
